# Transdiagnostic Mobile Health: Smartphone Intervention Reduces Depressive Symptoms in People With Mood and Psychotic Disorders

**DOI:** 10.2196/13202

**Published:** 2019-04-12

**Authors:** Dror Ben-Zeev, Benjamin Buck, Phuonguyen Vu Chu, Lisa Razzano, Nicole Pashka, Kevin A Hallgren

**Affiliations:** 1 Behavioral Research In Technology and Engineering Center Department of Psychiatry and Behavioral Sciences University of Washington Seattle, WA United States; 2 Health Services Research and Development VA Puget Sound Healthcare System Seattle, WA United States; 3 Department of Health Services School of Public Health University of Washington Seattle, WA United States; 4 Center on Mental Health Services Research and Policy Department of Psychiatry University of Illinois at Chicago Chicago, IL United States; 5 Thresholds Inc Chicago, IL United States

**Keywords:** mHealth, schizophrenia, bipolar disorder, depression, illness management, symptoms, transdiagnostic

## Abstract

**Background:**

Depression is the most prevalent mental health problem. The need for effective treatments for depression far outstrips the availability of trained mental health professionals. Smartphones and other widely available technologies are increasingly being leveraged to deliver treatments for depression. Whether there are patient characteristics that affect the potency of smartphone interventions for depression is not well understood.

**Objective:**

This study aimed to evaluate whether patient characteristics including clinical diagnosis, depression severity, psychosis status, and current use of antidepressant medications impact the effects of an evidence-based smartphone intervention on depressive symptoms.

**Methods:**

Data were collected as part of a 2-arm randomized controlled trial comparing a multimodal smartphone intervention called FOCUS with a clinic-based intervention. Here, we report on 82 participants assigned to 12 weeks of FOCUS treatment. We conducted assessments of depressive symptoms using the Beck Depression Inventory-second edition (BDI-II) at baseline, postintervention (3 months), and follow-up (6 months). We tested for differences in the amount of improvement in BDI-II scores from baseline to posttreatment and 6-month follow-up between each of the following patient subgroups using 2 (group) × 2 (time) mixed effects models: diagnosis (ie, schizophrenia spectrum disorder vs bipolar disorder vs major depressive disorder), depression severity (ie, minimal-mild vs moderate-severe depression), psychosis status (ie, presence vs absence of psychotic symptoms), and antidepressant use (ie, taking antidepressants vs not taking antidepressants).

**Results:**

The majority of participants were male (60%, 49/82), African American (65%, 53/82), and middle-aged (mean age 49 years), with a high school education or lower (62%, 51/82). There were no differences in patient demographics across the variables that were used to stratify the analyses. There was a significant group × time interaction for baseline depression severity (*F*_1,76.8_=5.26, *P*=.02 [posttreatment] and *F*_1,77.4_=6.56, *P*=.01 [6-month follow-up]). Participants with moderate or severe depression had significant improvements (*t*_42_=3.20, *P*=.003 [posttreatment] and *t*_42_=4.20, *P*<.001 [6-month follow-up]), but participants with minimal or mild depression did not (*t*_31_=0.20, *P*=.84 [posttreatment] and *t*_30_=0.43, *P*=.67 [6-month follow-up]). There were no significant group × time interactions for diagnosis, psychosis status, or antidepressant medication use. Participants with minimal or mild depression had negligible nonsignificant improvements (<1 point on the BDI-II). Reduction in depression in all other groups was larger (range 1.7-6.5 points on the BDI-II).

**Conclusions:**

Our results suggest that FOCUS can be deployed to treat moderate to severe depressive symptoms among people with schizophrenia spectrum disorders, bipolar disorder, and major depressive disorder, in concert with antidepressant medications or without them, in both people with and without active psychotic symptoms. The study results are consistent with research on transdiagnostic models in psychotherapy and extend our knowledge about the potential of transdiagnostic mobile health.

**Trial Registration:**

ClinicalTrials.gov NCT02421965; http://clinicaltrials.gov/ct2/show/NCT02421965 (Archived by WebCite at http://www.webcitation.org/76pyDlvAS)

## Introduction

### Background

Depression is a universal experience. Symptoms of depression are the most common mental health concern reported across nationalities, ethnicities, and age groups worldwide [1,2]. Depression is prevalent in all clinical settings, either as the primary issue that brings people to seek mental health care [3,4] or as a significant comorbid concern that emerges in those contending with medical illnesses [5-8], physical disabilities [9,10], relationship problems [11], work and educational difficulties [12], or substance use problems [13]. Depression is a source of enormous financial and societal burden. It is the second leading cause of years lived with disability worldwide [14], with overall medical and job loss costs estimated at US $210.5 billion annually [15]. It is also implicated in half to two-thirds of all completed suicides [16]. The need for effective treatments for depression far outstrips the availability of resources that can be delivered by trained mental health professionals. This tension has led to the emergence of new models of care that are no longer dependent on the availability of highly trained mental health specialists, including integration of depression treatments into the offerings of primary care settings [17-19], training of paraprofessionals and family members to provide support [20], and using new technology to expand the breadth and reach of depression management resources [21,22].

It is only fitting that in recent years the most common mental health problem is starting to be addressed with the aid of one of the most widely used technologies on the planet: the mobile phone [23,24]. Smartphones—contemporary mobile phones with multimedia players, internet connectivity, and the ability to host apps—are increasingly being leveraged to deliver treatments for depression. A recent meta-analysis of 18 randomized controlled trials (RCTs) of smartphone-based mental health interventions for depressive symptoms found that these treatments had positive effects in comparison with both active interventions and inactive control conditions. Therapeutic effects were found for those with self-reported mild to moderate depression but were not seen among those with diagnoses of major mood disorders [25]. The authors of the meta-analysis outlined that these findings may be linked to the small and underpowered subsample sizes used in the studies involving those with clinical diagnoses and emphasized the need for further research to deepen our understanding of which populations stand to benefit the most from smartphone interventions for depressive symptoms. Digital treatments are very novel in mental health care, and there is still uncertainty about whether these approaches are appropriate for all patients [26,27]. Research evaluating the effects of mobile health (mHealth) on people with both mild and more severe symptomatology can address this gap in our knowledge.

### Objectives

Given that depressive symptoms commonly emerge in many forms of psychopathology [28], in this study, we examined the effects of an evidence-based smartphone intervention called FOCUS on depressive symptoms transdiagnostically among people with mood and psychotic disorders. We evaluated whether several patient clinical characteristics (ie, diagnosis, depression symptom severity, psychosis symptoms, and antidepressant medication use) impact the effects of the smartphone intervention on depressive symptoms in an RCT.

## Methods

### Study Descriptions

We conducted an assessor-blind, 2-arm, RCT between 2015 and 2017. The project was conducted in partnership with a large Chicago-based mental health agency that provides services to a range of people with psychiatric conditions. The study was approved by the Institutional Review Boards of the University of Washington and Dartmouth College and monitored continuously by an independent safety monitoring board. All study participants completed informed consent. Individuals were randomized (1:1 ratio) into 1 of the 2 treatment arms: an mHealth intervention delivered via smartphone (FOCUS) or a clinic-based group intervention (Wellness Recovery Action Plan). Interventions were deployed for a period of 12 weeks. We conducted assessments at baseline, postintervention (3 months), and follow-up (6 months). Participants were compensated $30 per assessment. The study was registered in ClinicalTrials.gov, and the main RCT comparison outcomes were reported in an earlier publication [29]. Here, we conducted a secondary analysis focusing specifically on patients who received the FOCUS smartphone intervention, examining whether several patient clinical characteristics preintervention affected the impact of the intervention on their depressive symptoms.

### Smartphone Intervention

FOCUS is a multimodal, smartphone-delivered intervention that was originally designed to support the recovery of people with schizophrenia [30] but has since been deployed among multiple diagnostic groups [29,31,32]. The FOCUS intervention comprises a FOCUS app that is used independently by patients, a Web-based clinician dashboard that summarizes participants’ responses to self-assessments and their use of various FOCUS resources, and an mHealth support specialist who helps participants make meaningful use of the FOCUS intervention and provides technical troubleshooting assistance via brief weekly phone calls [32]. FOCUS treatment content targets 5 broad domains: mood (ie, depression and anxiety), voices (ie, auditory hallucinations), sleep problems, medication use, and social functioning. Content takes the form of either brief video, audio, or sequences of digital screens with written material coupled with visual displays. The FOCUS app includes preprogrammed daily prompts (questions that take over the home screen), followed by tailored intervention content. Participants who identify as having significant difficulties with depression at baseline may be assigned *mood* focused prompts, but all content is accessible on demand to all users 24/7 without restriction.

### Participants

A total of 82 participants who were assigned to the FOCUS smartphone arm in the RCT are included in this report; posttreatment and 6-month follow-up data were available for 75 (91.5%) and 74 (90.2%) participants, respectively. Participants were identified by research staff and clinical teams at 3 agency sites. Study inclusion criteria included the following: chart diagnosis of schizophrenia, schizoaffective disorder, bipolar disorder, or major depressive disorder; aged 18 years or older; and a rating of “3” or lower on 1 of the 3 items comprising the *Domination by Symptoms* factor from the Recovery Assessment Scale [33]. Exclusion criteria included hearing, vision, or motor impairment (ie, that could affect the operation of a smartphone); less than 5th-grade English reading ability (per the Wide Range Achievement Test-4) [34]; and exposure to study interventions in the past 3 years. Participants continued to be eligible for all other clinical services including crisis intervention, assertive community treatment, supported employment, psychiatric evaluation, medication monitoring, psychosocial rehabilitation, and case management. Services were delivered in-person in the community or at 1 of the agency’s multiple locations.

### Measures

The primary outcome (depression symptoms) was measured with the Beck Depression Inventory-second edition (BDI-II) [35]. The BDI-II is a self-report questionnaire with 21 items rated on a 4-point scale that can be summed for a continuous total depression severity score ranging from 0 to 63; scores can also be categorized to characterize symptom severity (0-13=minimal, 14-19=mild, 20-28=moderate, and 29-63=severe). For subgroup analyses, participants were categorized based on whether they had minimal to mild depression versus moderate to severe depression at baseline. Psychotic symptoms were assessed with the Psychotic Symptom Rating Scales (PSYRATS) [36], a semistructured interview instrument that assesses the severity of auditory hallucinations (eg, frequency, duration, loudness, and distress) and delusions (eg, preoccupation, conviction, and disruption). The PSYRATS comprises 17 items, each rated on a 4-point scale and summed for a total psychotic symptoms score. Given the distribution of psychotic symptoms at baseline in our sample (63.8% endorsing none), we dichotomized our sample based on whether individuals had any psychotic symptoms (vs none) at baseline. Antidepressant medication use was recorded by study assessors during baseline interviews and follow-up calls where participants were asked to read their medication labels to study staff. We dichotomized our sample based on whether study participants were actively taking any antidepressant medications (vs not) before commencing study interventions. Participants’ diagnosis was recorded from the electronic health records. Diagnoses are determined by licensed clinical social workers or licensed clinical professional counselors who interview clients about their mental health challenges and history, examine any prior medical records, and consult with the agency’s medical director who is a board-certified psychiatrist.

### Data Analytic Plan

We conducted a series of 2 (group) × 2 (time) mixed effects models to evaluate whether there were differences in the amount of clinical improvement in BDI-II scores between each of the following groups: (1) Diagnosis, schizophrenia spectrum disorder versus bipolar disorder versus major depressive disorder; (2) Depression severity, minimal-mild versus moderate-severe depression; (3) Psychosis symptoms, presence versus absence of psychotic symptoms, and (4) Antidepressant medications, taking antidepressants versus not taking antidepressants. Any significant group × time interactions would indicate that the amount of clinical improvement was moderated by the baseline group variable; interactions that were significant were followed by paired sample *t* tests to evaluate the significance of the amount of changes in BDI-II scores within each specific group.

## Results

### Demographics and Study Variables

Descriptive statistics are presented in Table 1. The majority of participants were male and African American; the mean age was 49 years. Most participants had a high school education or lower (62%, 51/82) and had used a smartphone before entering the study (73%, 60/82). The 3 diagnostic categories specified as inclusion criteria (schizophrenia, schizoaffective disorder, bipolar disorder, and major depressive disorder) were well represented within the sample. There were no differences in patient demographics across the baseline measures that were used to stratify our analyses (diagnosis, depression severity, psychosis symptoms, and antidepressant medications).

**Table 1:** Descriptive statistics at baseline (N=82).

Demographic and study variables		Statistics
Age (years), mean (SD)		49 (10.1)
Male, n (%)		49 (60)
Previously used smartphone, n (%)		60 (73)
**Race, n (%)**		
	White	22 (27)
	African American	53 (65)
	Other or more than 1 race	7 (9)
**Education, n (%)**		
	High school or less	51 (62)
	More than high school	31 (38)
**Diagnoses, n (%)**		
	Schizophrenia/schizoaffective disorder	38 (46)
	Bipolar disorder	21 (26)
	Major depressive disorder	23 (28)
**Depression, n (%)**		
	Minimal or mild	35 (43)
	Moderate or severe	47(57)
**Psychosis symptoms, n (%)**		
	Absent	51 (62)
	Present	31 (38)
**Antidepressant medications, n (%)**		
	None	34 (41)
	One or more	46 (56)
	Unknown	2 (2)

### 6-Month Follow-Up and Posttreatment Results

**Figure 1:**
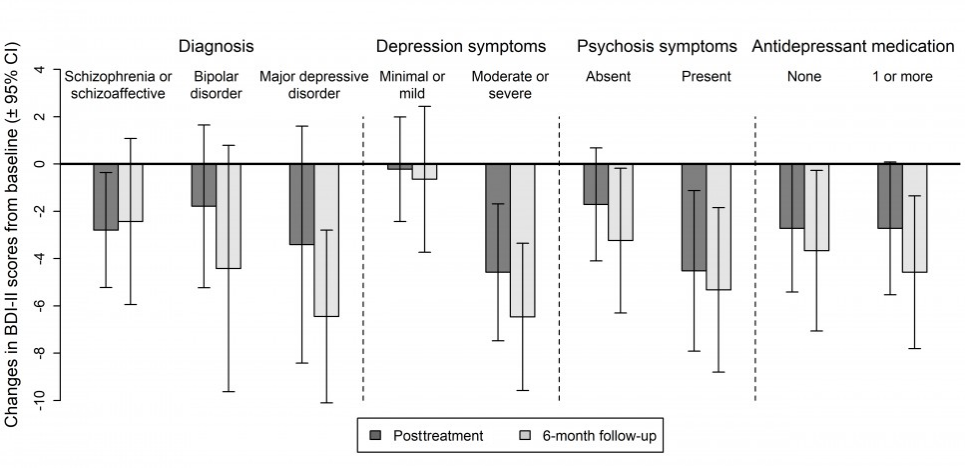
Changes in BDI-II edition depression scores by patient characteristics at baseline. BDI-II: Beck Depression Inventory-second edition.

As was also reported in the parent trial [29], participants in the FOCUS condition had significant reductions in BDI-II scores at posttreatment (mean change=–2.72; *t*_74_=–2.80; *P*=.006) and 6 months (mean change=–4.03; *t*_73_=–3.53; *P*<.001) over baseline, indicating that for the full sample, depression symptoms improved during the FOCUS intervention and that these improvements were maintained at the 6-month follow-up. There were no significant group × time interactions for diagnostic group (*F*_2,73.8_=0.16, *P*=.86 [posttreatment] and *F*_2,74.5_=1.07, *P*=.35 [6-month follow-up]), psychosis symptoms (*F*_1,75.1_=1.89, *P*=.17 [posttreatment] and *F*_1,75.0_=0.70, *P*=.41 [6-month follow-up]), or antidepressant medication use (*F*_1,77.3_<0.01, *P*=.95 [posttreatment] and *F*_1,80.0_=0.20, *P*=.65) [6-month follow-up]), indicating that there were no significant differences in the amount of improvement participants experienced over time between the subgroups that were defined by these 3 baseline variables. However, there was a significant group × time interaction for baseline depression severity (*F*_1,76.8_=5.26, *P*=.02 [posttreatment] and *F*_1,77.4_=6.56, *P*=.01 [6-month follow-up]), indicating that the amount of improvement in depression scores was different between participants with minimal or mild depression symptoms compared with participants with moderate or severe depression symptoms. Follow-up tests within these groups indicated that participants with minimal or mild depression did not have significant reductions in depression symptoms from baseline to posttreatment (difference=–0.22; *t*_31_=–0.20; *P*=.84) or follow-up (difference=–0.65; *t*_30_=–0.43; *P*=.67); however, participants with moderate or severe depression did have significant reductions in depression symptoms at posttreatment (difference=–4.58; *t*_42_=–3.20; *P*=.003) that were also maintained at follow-up (difference=–6.57; *t*_42_=–4.20; *P*<.001). Average levels of change within all subgroups (with 95% CIs) are further characterized (see Figure 1).

## Discussion

### Principal Findings

The results of this study deepen our understanding of patient clinical characteristics that may impact the effectiveness of FOCUS on their level of depression, independent of their assigned diagnostic label. We found that (1) FOCUS produced significant and sustained (at 6-month follow-up) reduction in depression among people who had moderate to severe depressive symptoms, effects that were not seen among people with minimal to mild depressive symptoms; (2) FOCUS produced significant and sustained reductions in depression among people with schizophrenia/schizoaffective disorder, bipolar disorder, and major depressive disorder; (3) FOCUS produced significant and sustained reductions in depression among people with psychotic symptoms and among people without any indication of psychosis; and (4) FOCUS produced significant and sustained reduction in depression in both people who were taking antidepressant medications and people who were not.

Our findings suggest that FOCUS might be a useful intervention to address moderate to severe depressive symptoms among individuals with an array of mental illnesses. Depressive symptoms are common among people experiencing psychosis [37], are linked with poorer outcomes [38,39], and often persist or recur even with antidepressant treatment [40]. Our findings go against current skepticism about the viability of computerized interventions for people with psychosis [26] as FOCUS produced significant positive effects in both individuals with and without active psychotic symptoms and in both people with and without a schizophrenia spectrum diagnosis.

In the context of precision medicine in mental health care [41] and the growing interest in customization of treatment for well-defined populations, this study can inform practical clinical decision making. Our results suggest that FOCUS can be deployed to effectively treat depression transdiagnostically among people with moderate to severe depressive symptoms, in concert with antidepressant medications or without them, in both people with and without co-occurring psychotic symptoms.

### Limitations

The study has several limitations. First, because mHealth was adjunctive to existing service provided through the community agency, other services may also have contributed to the positive changes that occurred during the study period. Second, the original study was designed with sufficient power to detect treatment changes in the overall sample, and thus, the subgroup analyses presented here should be interpreted with caution. Finally, dichotomous groups based on baseline variables were broad; a larger sample would allow examination of more fine-grained or continuous relationships between demographic or clinical characteristics and treatment benefit.

### Conclusions and Future Directions

FOCUS was designed to maximize accessibility for those who are most impaired [30] while targeting several domains that are relevant transdiagnostically. Multicomponent mHealth systems are needed for users who may have diverse and evolving cognitive, emotional, and behavioral challenges. The study results are consistent with research on transdiagnostic models in clinic-based psychotherapy [42] and computerized interventions [43,44] and extend what we know about transdiagnostic mHealth. The findings also contribute to our growing awareness that mental health difficulties are multidimensional [45]. As we uncover more about heterogeneity within clinical conditions and advance our understanding of dimensionality in psychopathology, we will increasingly move away from categorical conceptualizations of “healthy” versus “ill” and “diagnosis A” versus “diagnosis B” [46]. Our mHealth interventions will likely follow suit, and like FOCUS, will continue to evolve into multidimensional and multicomponential systems [47,48]. As such, mHealth will become a more versatile mental health management approach that can serve a broader spectrum of needs.

## Abbreviations

BDI-II: Beck Depression Inventory-second edition
mHealth: mobile health
PSYRATS: Psychotic Symptom Rating Scales
RCT: randomized controlled trial
